# ATP control of dynamic P1 ParA–DNA interactions: a key role for the nucleoid in plasmid partition

**DOI:** 10.1111/j.1365-2958.2010.07314.x

**Published:** 2010-08-18

**Authors:** Anthony G Vecchiarelli, Yong-Woon Han, Xin Tan, Michiyo Mizuuchi, Rodolfo Ghirlando, Christian Biertümpfel, Barbara E Funnell, Kiyoshi Mizuuchi

**Affiliations:** 1 Department of Molecular Genetics, University of TorontoToronto, Ontario M5S 1A8, Canada; 2 Laboratory of Molecular Biology, National Institute of Diabetes, and Digestive and Kidney Diseases, National Institutes of HealthBethesda, MD 20892-0540, USA

## Abstract

P1 ParA is a member of the Walker-type family of partition ATPases involved in the segregation of plasmids and bacterial chromosomes. ATPases of this class interact with DNA non-specifically *in vitro* and colocalize with the bacterial nucleoid to generate a variety of reported patterns *in vivo*. Here, we directly visualize ParA binding to DNA using total internal reflection fluorescence microscopy. This activity depends on, and is highly specific for ATP. DNA-binding activity is not coupled to ATP hydrolysis. Rather, ParA undergoes a slow multi-step conformational transition upon ATP binding, which licenses ParA to bind non-specific DNA. The kinetics provide a time-delay switch to allow slow cycling between the DNA binding and non-binding forms of ParA. We propose that this time delay, combined with stimulation of ParA's ATPase activity by ParB bound to the plasmid DNA, generates an uneven distribution of the nucleoid-associated ParA, and provides the motive force for plasmid segregation prior to cell division.

## Introduction

Partition is an essential process that ensures stable inheritance of genetic material. Cells have developed several different mechanisms to efficiently move chromosomal DNA to proper cellular locations prior to cell division, and the same is true for low-copy-number bacterial plasmids. Plasmids are useful models for investigating these intricate processes in prokaryotes, as they typically encode only three essential partition machinery components: a *cis*-acting partition site, a partition site binding protein and a partition ATPase (Slavcev and Funnell, 2004). The P1 plasmid is a stable low-copy-number plasmid in *Escherichia coli*. Its partition site and partition site binding protein are *parS* and ParB respectively. ParA, the ATPase, has two known functions, which are modulated by the type of adenine nucleotide bound (Bouet and Funnell, 1999). The ADP form of ParA regulates the expression of ParA and ParB through site-specific DNA binding to the *par* operator. However, the operator-binding activity of ParA is not directly required for the partition reaction itself. ParA–ATP is thought to act in the physical segregation of plasmids. *In vivo*, hydrolysis-deficient ParA mutants are defective for partition (Davis *et al*., 1996; Fung *et al*., 2001), and *in vitro*, a ParA interaction with the partition complex is ATP-dependent (Bouet and Funnell, 1999). However, the role of nucleotide hydrolysis by ParA in plasmid partition and the mechanism of plasmid localization are unknown.

Chromosomal and plasmid partition systems have been categorized according to the nature of the ATPase involved (Gerdes *et al*., 2000). Type I partition systems utilize a specific variant of Walker-type ATPases, and include P1 *par*, F plasmid *sop* and all chromosomal *par* systems. Bacterial MinD ATPases, which also impart positional information (septum localization), are related to this family. The other major class of partition systems, Type II, such as the *par*MRC system of R1 plasmid, encode ATPases that show structural similarity to eukaryotic actin. Type II ATPases function by forming filaments that display ATP-dependent dynamic instability and push plasmids apart in the cell (Moller-Jensen *et al*., 2003; Garner *et al*., 2007). Several Walker-type partition ATPases have also been observed to undergo ATP-dependent polymerization, but how ParA polymerization contributes to partition has not been established and several different models have been suggested for this class of partition ATPases (Barillà*et al*., 2005; Lim *et al*., 2005; Ebersbach *et al*., 2006; Bouet *et al*., 2007; Pratto *et al*., 2008).

One striking difference between the two system types is the non-specific DNA-binding activity observed for a number of Walker-type ATPases. In general, the ATPase activity of these proteins is synergistically stimulated by DNA and by their cognate ParB *in vitro* (Davis *et al*., 1992). *In vivo*, dynamic oscillatory behaviour has been observed with fluorescent variants of some Walker-type partition ATPases (Marston and Errington, 1999; Quisel *et al*., 1999; Ebersbach and Gerdes, 2004; Fogel and Waldor, 2006; Hatano *et al*., 2007; Pratto *et al*., 2008). These resemble the dynamics of MinD-GFP (Raskin and deBoer, 1999), except that unlike MinD oscillations, which traverse the entire cell length on the membrane, the dynamic patterns of partition ATPases are restricted to the nucleoid region. The behaviour of several ParA mutants suggests that the DNA-binding activity of Walker-type ATPases plays an essential role in partition. An F SopA mutant that is deficient in non-specific DNA binding, but retains its specific *sop* promoter-binding activity, displays a severe partition defect *in vivo* (Castaing *et al*., 2008). A set of conserved arginines in Soj, a *Bacillus subtilis* ParA homologue, is essential for both nucleoid binding and plasmid stability *in vivo* (Hester and Lutkenhaus, 2007). Finally, cell biology experiments have shown that plasmids colocalize with bacterial nucleoids and this colocalization requires ParA (Erdmann *et al*., 1999; Ebersbach and Gerdes, 2004; Ringgaard *et al*., 2009). Thus, a ParA–non-specific DNA interaction is presumed to be critical for the partition reaction but mechanistic insight is lacking.

In this study we investigate how ATP promotes and regulates the DNA-binding activity of Walker-type partition ATPases. We used total internal reflection fluorescence microscopy (TIRFM) to visualize the interaction between DNA and a fluorescent variant of P1 ParA. We find that ParA's non-specific DNA-binding activity is highly dynamic and specifically requires ATP. We performed a series of kinetic and conformational analyses to identify the form of ParA responsible for DNA binding. We found that ParA gains competence for non-specific DNA binding after an ATP-dependent multi-step conformational change that precedes hydrolysis. A ParA mutant that binds ATP but cannot undergo this conformational change cannot bind non-specific DNA and is deficient for partition function *in vivo*. We propose that P1 plasmid localization requires a critical ATP-dependent interaction between ParA and the host bacterial chromosome, similar to the ATP-dependent interaction between MinD and the bacterial membrane. We present a model of plasmid partition that uses a variation of the reaction/diffusion type mechanism to explain plasmid positioning in the bacterial cell cycle.

There is mounting evidence that similar Walker-type ATPase-dependent patterning events are likely to be used in several other processes including: Type IV secretion (VirC1; Atmakuri *et al*., 2007), chemotaxis (PpfA; Thompson *et al*., 2006) and carbon fixation (ParA; Savage *et al*., 2010). We predict that these processes, and others, have evolved to exploit the nucleoid or membrane for positional information, and their localization events are dependent on a timing mechanism that is intrinsic to the ATPase.

## Results

### ParA binds DNA non-specifically in the presence of ATP

To study the interaction dynamics between P1 ParA and non-specific DNA in real time, we constructed and purified a functional ParA protein fused at its C-terminus to green fluorescent protein (GFP) (Fig. S1). We used TIRFM to visualize ParA binding to phage λ-DNA molecules that were tethered at one end to the flow cell surface (Greene and Mizuuchi, 2002). Buffer flow extended and confined the DNA molecules, along with bound ParA–GFP, within the evanescent illumination.

**Fig. 1:**
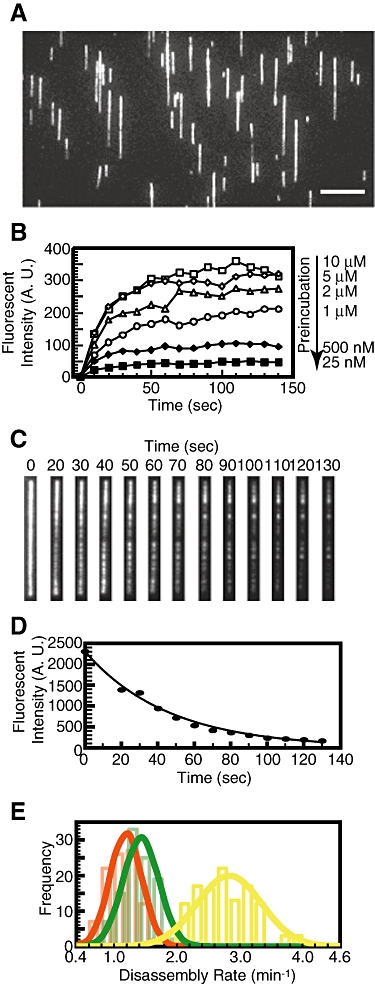
ParA–DNA interaction dynamics observed by TIRF microscopy.A. Representative image of ParA assembly. Scale bar indicates 10 µm.B. ParA was pre-incubated at the indicated concentrations with 1 mM ATP for 15 min and diluted to 25 nM ParA before infusion. Total fluorescence signal from single-tethered DNA molecules was plotted as a function of time.C. Measurement of ParA dissociation rate constant for individual DNA molecules. Time-lapsed fluorescence images of an individual λ DNA molecule.D. Corresponding ParA dissociation curve based on the fluorescent intensity in (C), with a single-exponential fit.E. Rate constant distribution histograms of ParA disassembly in plain buffer (green), buffer containing 1 mM ATP (red) and buffer containing 0.5 mg ml^−1^ sonicated salmon sperm DNA (yellow) with Gaussian fit.

When a buffer containing ParA–GFP was infused into the flow cell with ATP and Mg^2+^, ParA–GFP assembled onto DNA rapidly (Fig. 1A). In the absence of ATP, no DNA binding was detected. At different concentrations of ParA–GFP, the apparent steady-state level of ParA–GFP bound to DNA changed accordingly. Above 100 nM, the DNA was quickly saturated. At 10–50 nM, partial covering of DNA by ParA–GFP was observed with rapid flickering of the fluorescence signal along each DNA molecule, indicating ongoing fast association and dissociation cycles of ParA–GFP molecules (Movies S1–S3). ParA–GFP formed discontinuous clusters along DNA, whose fluorescence intensity increased or decreased from one frame to the next by small quantities, indicating that the minimum ParA unit responsible for DNA binding was relatively small oligomers. Long continuous coverage of the DNA was only observed at near saturating concentrations of ParA–GFP. Below 50 nM ParA–GFP, the fluorescence signal at apparent steady state was less than proportional to the protein concentration, becoming undetectable below 10 nM in some experiments, indicating the involvement of a cooperative process (data not shown). In the experiments above, ATP was incubated with the final concentration of ParA for several minutes before DNA binding was initiated. We tested the effect of pre-incubating different concentrations of ParA with ATP followed by dilution to 25 nM ParA immediately prior to infusion to the flow cell (Fig. 1B, Movies S1 and S2). Pre-incubation at higher ParA concentrations greatly enhanced DNA binding, indicating the existence of a higher-order ParA concentration-dependent step prior to DNA binding.

The dissociation kinetics of ParA from DNA were investigated using the TIRFM approach. After ParA–GFP binding to DNA reached apparent steady state, the solution was switched to a ‘disassembly buffer’ (without ParA), and then the fluorescence intensity, integrated over individual DNA molecules, was monitored as a function of time (Fig. 1C). The fluorescence decrease fit a single exponential decay function and displayed an off-rate constant of 0.8–1.8 min^−1^ (Fig. 1D). The off-rate was slightly slower when ATP was in the disassembly buffer suggesting that loss of bound ATP by ParA could be a small contributing factor for the dissociation (Fig. 1E). Addition of competitor DNA in the disassembly buffer significantly accelerated the apparent dissociation rate (Fig. 1E), indicating that ATP-bound ParA engages in inter-segmental transfer between DNA sites at higher local DNA concentrations.

Next, we investigated the nucleotide cofactor specificity for ParA–DNA binding. ATP in the above experiments was replaced with AMP, ADP, ADP+Pi, AMP-PNP, ATPγS, ADP-AlF_4_, ADP-BeF_x_ or ADP-VO_4_. None of the nucleotide cofactors tested except ATP supported ParA–DNA binding (data not shown). All tested adenosine di- and tri-phosphate analogues inhibited DNA binding in the presence of ATP, indicating that ParA could bind these nucleotides (data not shown). The DNA binding observed here therefore is distinct from, and does not reflect the site-specific DNA-binding activity of P1 ParA (to the par operator) that might occur at a lower sequence specificity: if so, ADP and ATP analogues would support DNA binding (Davey and Funnell, 1994). Thus, the ParA conformation necessary for non-specific DNA binding is highly specific for ATP. What might be the reason for this stringent nucleotide specificity?

### ATP hydrolysis is not a prerequisite for DNA binding

**Fig. 2:**
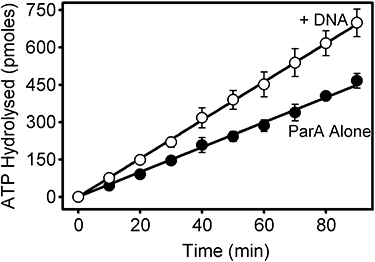
Pre-steady-state ATP hydrolysis kinetics of ParA. ParA and [γ-^32^P]-ATP (10 µM and 1 mM final concentrations respectively) were rapidly mixed in buffer A in the presence (empty circles) or absence (filled circles) of 100 µg ml^−1^ sonicated salmon sperm DNA. The hydrolysis product was measured after the indicated reaction time at 23°C.

We considered a possibility that the conformation of ParA competent for DNA binding is reachable only through the ATP hydrolysis step, and not when the reaction is started with the hydrolytic products (ADP with or without P_i_ analogues). Thus, non-hydrolysable ATP analogues would not function. Since the steady-state rate of ATP turnover by ParA is slow (0.017–0.025 min^−1^ at 23°C, see below), this proposal raises the possibility that the hydrolysis step is fast and the product release step is slow, resulting in accumulation of a post-hydrolysis state such as a special ADP-bound form of ParA. In this case, pre-steady-state kinetics of ATP hydrolysis should exhibit an initial burst phase. However, ParA did not display any sign of an initial burst. No significant deviation from the steady-state rate of hydrolysis was observed in the single turnover period. The presence of DNA slightly increased the steady-state rate of hydrolysis but again no burst phase was observed (Fig. 2). Thus, the product release step is not rate-limiting in the ATPase cycle, and ParA bound to its hydrolytic products must be a minor fraction of the enzyme population at steady state.

### ParA binds ATP in multiple steps

The rate of ParA dissociation from DNA in the presence of ATP (Fig. 1E) is much faster than the steady-state rate of ATP hydrolysis, which indicates that the dissociation step of ParA from DNA is unlikely to be coupled to any of the steps in the chemical ATPase cycle. This observation further points to an event upstream of hydrolysis to be responsible for licensing ParA to bind DNA. Therefore, we next focused on the ATP binding process. We first monitored nucleotide binding by ParA by making use of the fluorescent analogues MANT-ATP and MANT-ADP (Cremo and Yount, 1987). Both MANT-ATP and MANT-ADP were bound by ParA (Fig. S2). Although MANT-ATP did not support ParA–DNA binding (data not shown), MANT-ADP bound ParA with similar affinity as ADP with an apparent *K*_d_ of approximately 30 µM (Fig. S2C), and the change in fluorescence of MANT was a useful tool to detect initial nucleotide interactions with ParA. MANT–nucleotide binding kinetics were somewhat complex and took 20 s or longer to reach apparent steady state (Fig. S2A and E). The observed binding kinetics are slower than expected for simple nucleotide docking and suggests the involvement of a protein conformational step.

**Fig. 3:**
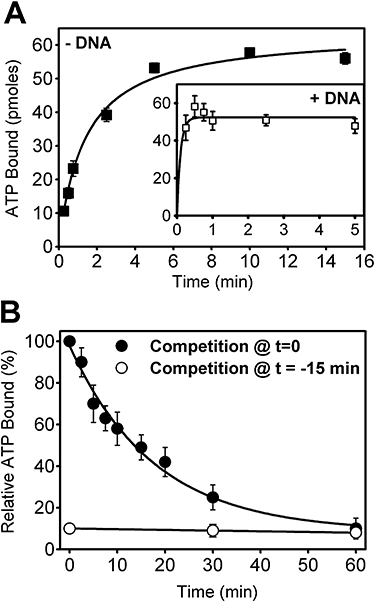
Kinetics of stable ParA–ATP complex formation and dissociation.A. ParA (2.5 µM, 75 pmoles) and 1 mM [α-^32^P]-ATP were mixed in 30 µl buffer A and after the indicated incubation time at 23°C, separated by a spin column (1 ml G-50 resin). Stably ParA-bound ATP excluded by the column was measured. The same experiment was carried out in the presence of 100 µg ml^−1^ sonicated salmon sperm DNA (inset).B. ParA (2.5 µM) and 0.1 mM [α-^32^P]-ATP were mixed in 30 µl of buffer A and after 15 min at 23°C, 2 mM unlabelled ATP was added. The sample was applied to a spin column after the indicated period and [α-^32^P]-ATP that remained stably bound to ParA was measured (filled circles). When 2 mM unlabelled ATP was added along with 0.1 mM [α-^32^P]-ATP prior to incubation (t = −15 min), no ATP binding was detected (empty circles).

Next, we measured ATP binding to ParA using spin columns to separate ParA-bound ATP from free ATP. This method specifically detects stable ParA–ATP complexes, which elute in the void volume (Fig. 3A). The amount of the stably bound ATP was proportional to and stoichiometric with ParA concentration (0.8 ATP:1 ParA) after ∼10 min of incubation. The observed pseudo-first-order association rate constant was 0.16 ± 0.02 min^−1^. To obtain the off-rate, [^32^P]-ATP was first incubated with ParA and then chased with an excess of unlabelled ATP. The amount of [^32^P]-ATP that remained bound to ParA was quantified as a function of time (Fig. 3B). The observed off-rate was 0.022 ± 0.002 min^−1^ and the half-life of the stable ParA–[^32^P]-ATP complexes was ∼14 min. This observation is similar to that reported for F SopA (Bouet *et al*., 2007). From the results reported below, we expected the kinetics of formation of the stably ATP-bound form of ParA to be faster in the presence of DNA. Spin-column experiments in the presence of DNA agreed with this prediction (Fig. 3A, inset). Formation of the stable, nucleotide-bound ParA species was unique to ATP, as it was not detectable using ADP (data not shown). The observed conversion rate to a stably ATP-bound form implicates an even slower conformational step for ParA–ATP binding. Therefore, we next sought to detect a nucleotide-induced conformational change in ParA.

### ParA undergoes an ATP-specific conformational change detectable by tryptophan fluorescence

**Fig. 4:**
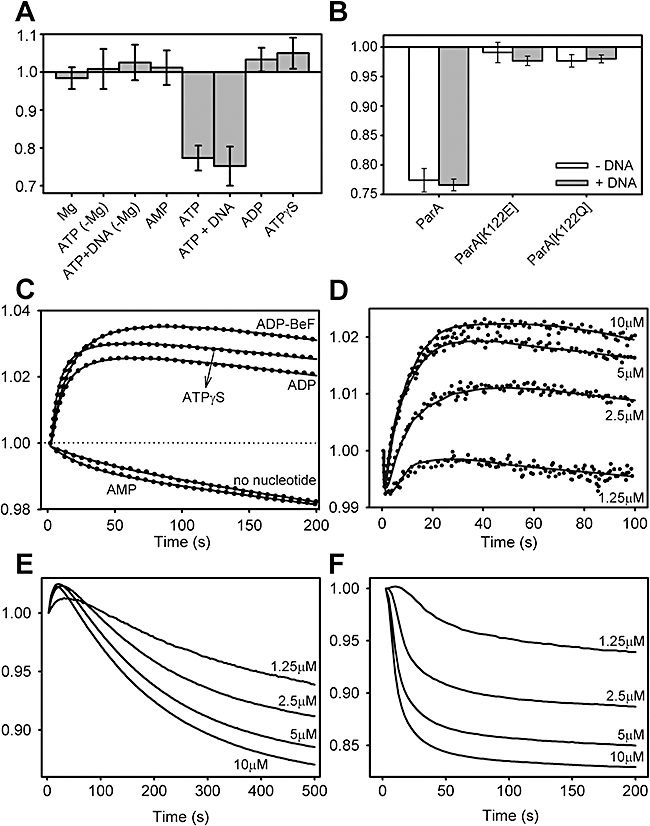
Nucleotide binding induces multi-step conformational changes of ParA.A. Steady-state tryptophan fluorescence change of ParA (5 µM) caused by the additives as indicated (1 mM nucleotide).B. Steady-state tryptophan fluorescence change of ParA variants (5 µM) in the presence of 1 mM ATP, with and without DNA.C. Kinetics of tryptophan fluorescence change of 5 µM ParA after addition of 1 mM nucleotide. (The slow decrease of fluorescence observed without nucleotide or with AMP is mostly due to fluorescence bleaching plus an effect of 2× dilution. Curves were not corrected for these effects.)D. ParA concentration dependence of the ADP-induced tryptophan fluorescence change.E. ATP induces an additional slow ParA tryptophan fluorescence change.F. The slow, ATP-specific change in ParA tryptophan fluorescence is accelerated by the presence of sonicated salmon sperm DNA (100 µg ml^−1^).For all panels, the *y*-axis is ParA tryptophan fluorescence intensity normalized to ParA tryptophan fluorescence prior to nucleotide addition, or the first data point in cases of stopped-flow experiments.

We have previously detected the structural effects of adenosine nucleotide binding by ParA using circular dichroism (Davey and Funnell, 1997). ParA displayed a slightly greater helicity when bound to ATP than when bound to ADP or ATPγS. To further investigate the structural change of ParA elicited by nucleotide binding, we measured tryptophan fluorescence changes. ParA contains one tryptophan at position 216, which is located away from the ATP binding site, but relatively close to the ADP-bound dimer interface (Dunham *et al*., 2009). We first measured ParA tryptophan fluorescence at steady state to identify which cofactors promoted a conformational transition that can be monitored by this method (Fig. 4A). In the presence of Mg^2+^ and ATP, ParA tryptophan fluorescence decreased approximately 20% relative to ParA without nucleotide. ADP and ATPγS induced a small but reproducible increase in fluorescence. In the presence of AMP, no significant change was observed. Tryptophan fluorescence measurements from all controls lacking Mg^2+^ or nucleotide were similar to ParA alone. These results confirm that ParA undergoes an ATP-specific conformational change.

We next tested whether DNA binding elicited any further conformational changes. The addition of DNA along with ATP did not noticeably alter fluorescence compared with ATP alone (Fig. 4A). Therefore after the ATP-dependent transition, ParA does not undergo additional structural transitions upon binding DNA that are detectable by this method. We hypothesized that the stably ATP-bound state, the lower tryptophan fluorescence state (ParA*:ATP), and the DNA-binding competent state are one and the same, and transition to this ParA form is slow due to a high-activation-energy barrier, which the non-hydrolysable ATP analogues are unable to overcome.

### ADP and ATP analogues induce a ParA conformational change

We next examined the kinetics of the small fluorescence increase with ADP or ATPγS to evaluate if it represents an earlier unstable nucleotide complex as detected by MANT–nucleotide binding experiments. We tested AMP, ADP, ATPγS, ADP-BeF_x_, AMP-PNP and ADP-AlF_4_ (Fig. 4C; data not shown for AMP-PNP and ADP-AlF_4_). All except AMP induced a 3–4% increase in ParA tryptophan fluorescence that took 20–40 s, after which the fluorescence reached an apparent steady state. AMP had no significant effect compared with no nucleotide control. Neither the kinetics nor the extent of fluorescence increase was noticeably affected by the presence of DNA (data not shown).

The extent of the fluorescence change induced by ADP was dependent on ParA concentration; the reaction approached saturation above 5 µM ParA and the half-saturation concentration was approximately 2 µM (Fig. 4D). The kinetics of this conformational change are faster than the kinetics of forming the stably ATP-bound form, but similar to the MANT–nucleotide binding kinetics. ParA can dimerize with and without nucleotide, and hydrodynamic studies have shown that nucleotide binding shifts the monomer–dimer equilibrium towards dimer (Davey and Funnell, 1994; Dunham *et al*., 2009). Judged by the ParA concentration dependence of the ADP-induced fluorescence increase and the similarity of the kinetics to that of MANT-ADP binding, we conclude that nucleotide binding is coupled to the formation of a nucleotide-bound dimer, ParA_2_:ANP_2_, which explains the relatively slow binding kinetics.

The first few data points in Fig. 4D suggested an earlier event. Closer examination showed that there was a fluorescence decrease of ∼1% in the first 0.5 s after addition of either ADP, or to a lesser extent, ATP (Fig. S3A). Unlike the fluorescence increase, the initial fluorescence decrease was not affected by ParA concentration. Therefore, we tentatively interpret this fluorescence decrease to represent the initial weak nucleotide binding of one nucleotide to ParA, which likely is not saturated at the nucleotide concentrations used in the experiments.

### ATP binding induces an additional slow conformational change with higher-order ParA concentration dependence

When ATP was added to ParA, the tryptophan fluorescence signal indicated an additional conformational transition (Fig. 4E). In the first phase of transition (2–50 s), fluorescence increased several percent. This, we believe corresponds to the same conformational change induced by ADP and ATP analogues. The second phase (t ∼50 s onward), which was unique to ATP, displayed a very slow decrease in fluorescence, taking ∼10 min to reach an apparent steady state at the range of ParA concentrations tested here. Based on the similar slow kinetics and the nucleotide specificity, we propose that the second transition generates the stably ATP-bound state of ParA. We found that addition of non-specific DNA increased the rate of transition ∼10-fold, allowing ParA conformation to reach an apparent steady state within ∼1 min at all ParA concentrations tested (Fig. 4F), which parallels the effect of DNA on formation of the stable ATP-bound form of ParA (Fig. 3A). Notably, the extent of the fluorescence change was not dramatically different with or without DNA, consistent with steady-state measurements of tryptophan fluorescence.

Although the timescales of the second transition are similar at ParA concentrations between 1.25 and 10 µM, the observed extent of the change exhibited higher-order protein concentration dependence. In the presence of DNA, this dependence became even more pronounced. The acceleration of the transition by DNA was dependent on DNA concentration (or rather the protein/DNA ratio) (Fig. S3B). If as proposed this ATP-bound state, ParA*:ATP represents the DNA-binding competent form of ParA, the fact that DNA accelerates this transition indicates that the transition state for this conformational change must be able to interact with DNA at least transiently. The ATP hydrolysis kinetics showed the steady-state rate from the beginning (Fig. 2), indicating that the conformation change does not strongly affect the hydrolysis rate.

### ATP-bound ParA is in a dimeric state

Why does the apparent extent of the ATP-specific conformational change depend on the protein concentration? We first considered that this transition might represent a local equilibrium between higher oligomeric forms of ParA. For example, if ParA*:ATP were a higher-order oligomer such as a tetramer, while the precursor state, ParA:ATP, is a dimer and the two states were connected by a reversible transition, a local equilibrium between the higher oligomer and dimer would respond to changes in protein concentration. We examined this possibility from several angles. First, we analysed the oligomeric state of ParA under different conditions.

**Fig. 5:**
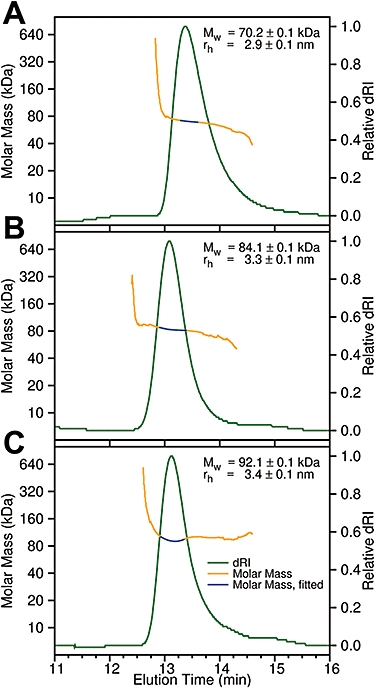
Oligomeric state of ParA in the presence and absence of nucleotide cofactors analysed by SEC/MALS.A. Size-exclusion column elution profile of ParA in the absence of any nucleotide.B. Same as (A), except ParA was pre-incubated with 0.5 mM ADP for 20 min and the column buffer contained 0.5 mM ADP.C. Same as (A), except ParA was pre-incubated with 0.5 mM ATP for 20 min and the column buffer contained 0.5 mM ATP.The elution profile monitored by relative differential refractive index (dRI) (green line) is shown together with the calculated molar mass (yellow line), of which the blue portion was used for the peak species calculation.

Results of tandem size-exclusion chromatography/multi-angle light scattering (SEC/MALS) analysis showed only dimers and no higher oligomeric forms were detected in the presence of ADP or ATP, in experiments carried out at a relatively high sample protein concentration of 30 µM (Fig. 5). In the absence of a nucleotide, the majority dimer fraction appeared to be in equilibrium with a small fraction of monomers judged by the broader and slightly shifted position of the peak and slightly smaller average molecular mass estimated. Dynamic light scattering experiments confirmed that no substantial amount of large aggregates is formed with or without added nucleotide (Fig. S5). Therefore, the basic unit of ParA*:ATP must be a dimer, ParA*_2_:ATP_2_.

Next, we investigated the reverse reaction of ParA*_2_:ATP_2_ formation, either by the addition of ADP as competitor, or looking for re-equilibration after twofold dilution (Fig. S4). We detected no evidence for reversal of the conformation prior to ATP hydrolysis. In the presence of ADP competitor, the conformational change reversal was observed at a very similar rate to that of the nucleotide exchange rate of the stable ATP-bound form of ParA (compare Fig. S4A with Fig. 3B). We conclude that the limited extent of ParA*_2_:ATP_2_ generation at lower ParA concentrations is not due to the local reversibility of the conformational change prior to ATP turnover, or because the stably ATP-bound form is obligatorily a large oligomer.

We propose that the structural transition proceeds through a short-lived intermediate that consists of multiple units of the precursor state ATP-bound dimers (ParA_2_:ATP_2_)_n_, without contribution of the product state dimers, ParA*_2_:ATP_2_. Thus, the structural transition becomes very slow when the precursor concentration drops below a critical level, because the product state population cannot assist the structural transition of the remaining population. Perhaps, this concentration-dependent step takes place prior to the transient DNA interaction thus explaining why the apparent extent of the reaction is not changed dramatically by DNA, but a later step is accelerated by DNA, thus the protein concentration-dependent step becoming the sole rate-limiting step of this transition in the presence of DNA. This also explains why the protein concentration dependence of the conformational change is more pronounced in the presence of DNA.

**Fig. 6:**
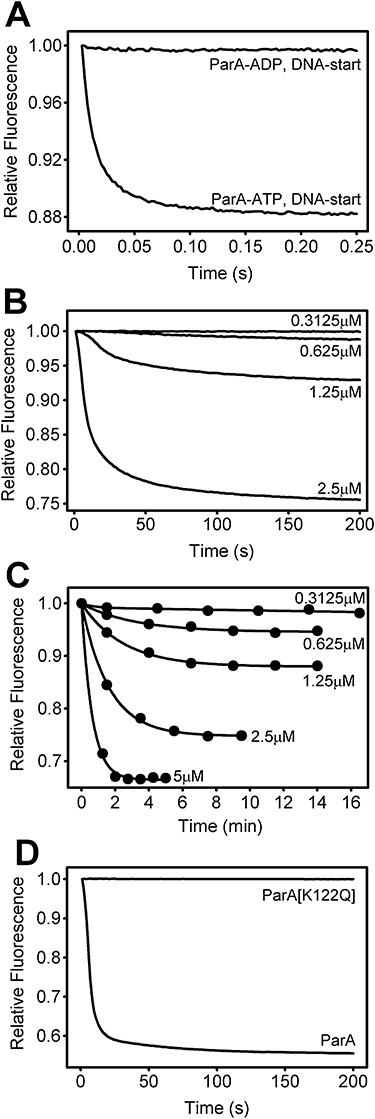
DNA binding kinetics of ParA.A. ParA (1.25 µM) was pre-incubated with 0.5 mM ATP or ADP for 15 min and rapidly mixed with 12.5 µg ml^−1^ fluorescence-labelled DNA, and fluorescence quenching by the bound protein was monitored (concentrations are final after mixing).B. ParA at indicated final concentrations was rapidly mixed with 12.5 µg ml^−1^ (final concentration) fluorescence-labelled DNA together with ATP.C. Kinetics of acquisition of DNA binding capacity by ParA upon addition of ATP in the absence of DNA.D. DNA binding by ParA and ParA[K122Q] (2.5 µM each) was measured as in (B).

The higher-order protein concentration dependence of the ATP-specific conformational transition affects consideration of another feature of the reaction kinetics, namely the sigmoidal time-course (Fig. 4E and F, also see Fig. 6B). The multi-step process of the ATP-induced conformational change described here easily explains the sigmoidality of the kinetics. It indicates that the reaction proceeds through multiple successive slow steps: in the absence of DNA, the rate of the second conformational transition is very slow, and the prerequisite nucleotide-induced dimerization is also slow. In the presence of DNA, as the second transition becomes faster, the earlier transition could become the sole rate-limiting step and then, the initial lag time would disappear. However, the higher-order concentration dependence of both transitions would preserve a significant lag time for the overall transition when the concentration of the precursor in the cytosol is low as expected for the physiological *in vivo* reaction where the major fraction of the protein is likely to be in the DNA-bound state. The ParA concentration dependence of the transition also suggests that *in vivo*, perhaps a minor but significant fraction of the ATP-bound ParA molecules might exist as ParA_2_:ATP_2_, which does not bind non-specific DNA, and could be the so-called ‘ADP-form’ that effectively binds the *par* operator.

### ParA*_2_:ATP_2_ is the DNA-binding competent state

We postulated that ParA*_2_:ATP_2_ is the state that is competent to bind non-specific DNA. To support this notion, we measured the kinetics of DNA binding by ParA following addition of ATP by using fluorescently labelled DNA. ParA bound to labelled and unlabelled DNA equally well as judged by competition experiments (Fig. S6). ParA binding to the labelled DNA resulted in significant quenching of the fluorescence signal, providing a real-time DNA binding assay.

First, we verified that DNA binding by ParA*_2_:ATP_2_ was indeed fast. When ParA was pre-incubated with ATP for 15 min, binding to labelled DNA occurred within approximately 20 ms (Fig. 6A). When ATP and DNA were simultaneously mixed with ParA, the DNA binding by ParA was much slower (Fig. 6B). This time-course is very similar to that of the formation of ParA*_2_:ATP_2_ in the presence of DNA (Fig. 4F). No difference in binding kinetics was observed if DNA was first pre-incubated either with ParA or with ATP (data not shown). DNA binding kinetics were measured at different ParA concentrations (Fig. 6B). Each time-course exhibited sigmoidal kinetics, and above 1µM ParA the reaction approached apparent steady state 20–50 s after ATP addition. Varying ParA concentrations at constant DNA concentration showed that the amount of DNA used was saturated at around 3 µM ParA, which corresponds to ∼6 bp/ParA monomer (Fig. 6C). If the fraction of ParA that binds to DNA were constant, every twofold reduction of the ParA concentration below the saturation concentration should have halved the extent of the observed fluorescence quenching. Instead, every twofold reduction of ParA concentration resulted in significantly more than a twofold reduction in DNA binding, indicating higher-order ParA concentration dependence of the extent of conversion to the DNA binding form as we predicted based on the tryptophan fluorescence experiments.

Next, we analysed the kinetics of generation of the DNA binding form of ParA after ATP addition in the absence of DNA. ParA and ATP were pre-incubated and, at intervals, mixed with the labelled DNA in a stopped-flow apparatus. The extent of DNA binding then observed in a 0.25 s time-course of stopped-flow measurement reflected the amount of ParA in the DNA-binding competent form that was generated during the pre-incubation. The results (Fig. 6C) demonstrate that the kinetics of this conformational change were slow, taking more than 5 min as predicted by the tryptophan fluorescence experiments. Thus, the kinetics of the DNA binding capacity gain by ParA agree very well with those predicted by the tryptophan fluorescence experiments. Finally, we confirmed that double-stranded DNA was the preferred substrate for this non-specific binding activity of ParA*_2_:ATP_2_; RNA and single-stranded DNA were poor competitors in this assay (Fig. S6). We conclude that the ParA conformation with stably bound ATP and reduced tryptophan fluorescence represents the DNA-binding competent state.

### ParA*_2_:ATP_2_ and non-specific DNA binding are required for partition

Mutation of the catalytic lysine residue conserved in the nucleotide binding site of ParA (K122) has provided insight into the activities of ParA (Fung *et al*., 2001). ParA[K122Q] is deficient for ATP hydrolysis and partition, but it is still competent in ATP binding and behaves as a ‘super-repressor’ of the *par* operon. *In vitro*, ParA[K122Q] can still bind DNA site-specifically to the *par* operator in the presence of ADP, ATP or ATP analogues. ParA[K122E], on the other hand, cannot bind or hydrolyse ATP, and is defective in both repressor and partition functions. We used these mutants to determine which *in vivo* function of ParA, partition or repression, was coupled to the formation of ParA*_2_:ATP_2_ and non-specific DNA-binding activity. Since ParA[K122E] cannot bind ATP, we expected and observed that it was unable to undergo the ATP-dependent conformational change as measured by changes in tryptophan fluorescence (Fig. 4B). ParA[K122Q], although able to bind ATP (Fung *et al*., 2001), also did not show any significant changes in tryptophan fluorescence in the presence of ATP, with or without added DNA (Fig. 4B). ParA[K122Q] was also unable to bind non-specific DNA in the presence of ATP (Fig. 6D), consistent with its inability to form ParA*_2_:ATP_2_. These results confirm that formation of ParA*_2_:ATP_2_ is necessary for ATP-dependent non-specific DNA-binding activity. Further, they argue that ParA[K122Q] is defective for partition activity because it cannot form ParA*_2_:ATP_2_ and cannot bind non-specific DNA.

## Discussion

### Nucleoid binding by ParA is driven by ATP

A family of Walker-type ATPases have important roles in chromosome and plasmid partition, but the underlying molecular mechanism has been elusive. We have identified a stable intermediate of P1 ParA, ParA*_2_:ATP_2_, which forms through a slow, ATP-specific conformational change that precedes ATP hydrolysis. Below is a summarized kinetic mechanism that illustrates this transition:


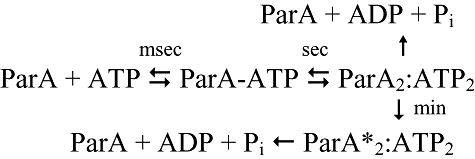


ParA_2_*:ATP_2_ can reversibly bind non-specific DNA prior to ATP hydrolysis. Mutation of the conserved lysine to glutamine in the Walker A ATP binding motif does not eliminate ATP binding by ParA but does eliminate the formation of ParA*_2_:ATP_2_ and non-specific DNA-binding activity *in vitro* and partition activity *in vivo*. We conclude that formation of ParA*_2_:ATP_2_ is a necessary step in the partition reaction.

A number of models for the action of Walker partition ATPases have been proposed. Dynamically unstable filament formation has been suggested following the example of actin-type partition ATPases and also based on the observations of ATP-dependent aggregation or polymerization of some of the members of this ATPase class (Suefuji *et al*., 2002; Barillà*et al*., 2005; Lim *et al*., 2005; Ebersbach *et al*., 2006; Bouet *et al*., 2007). We performed SEC/MALS on 30 µM ParA and we did not observe any evidence for ATP-dependent self-supporting filament formation by P1 ParA alone. ParA was diluted several fold as it passed through the size-exclusion column; however, the protein concentration was still substantially higher than the estimated *in vivo* concentration. At 40 µM, ParA has been observed to form ATP-dependent filaments by electron microscopy (Dunham *et al*., 2009), and we saw some higher oligomer when SEC was performed at 300 µM ParA (data not shown), but how this relates to ParA activity at physiological concentrations remains to be determined.

### Similarities between Par and Min localization systems

In this report, we examined the interaction dynamics between ParA, ATP, and non-specific DNA, an abundant material inside bacterial cells in the form of nucleoids. Non-specific DNA binding by partition ATPases has been noted in several systems (Bouet *et al*., 2007; Hester and Lutkenhaus, 2007), and has been suggested to form a scaffold on which ParA polymerization occurs (Ringgaard *et al*., 2009). However, biochemical evidence describing how polymerization and non-specific DNA binding cooperate in partition is still lacking. In addition, we believe it highly significant that this class of partition ATPases is more closely related to bacterial MinD ATPases than to actin-like ATPases. MinD ATPases also impart positional information in the cell, and this function requires its ATP-dependent membrane-binding activity. We propose that ParA's ATP-dependent DNA-binding activity is analogous to the membrane-binding activity of MinD, and based on our findings reported here, we propose a variation of the reaction-diffusion type mechanism that has been considered for the MinD system. Dynamic oscillation of MinD from cell-pole to cell-pole generates a time-averaged MinD distribution minimum at mid-cell (Raskin and deBoer, 1999). The initiation of cell division by FtsZ polymerization is limited to the mid-cell because MinC, which inhibits FtsZ polymerization, binds to and travels with MinD, so its concentration is lowest at mid-cell. Reaction-diffusion type models have been proposed to explain MinD oscillation, whose ATPase activity is controlled by MinE (Howard *et al*., 2001; Meinhardt and deBoer, 2001; Kruse, 2002; Huang *et al*., 2003; Drew *et al*., 2005). Since several Walker-type partition ATPases exhibit oscillatory behaviour, it is attractive to consider the mechanisms of these systems under the same light. ParB, which stimulates ParA ATPase activity, would play the analogous role of MinE in the partition version, with the help of *parS* DNA for organization into the partition complex. A time delay in MinD membrane re-binding has been considered as one of the critical elements in some of the models proposed (see below). We believe that the time-delay mechanism we found for ParA–DNA binding after ATP binding is one of the crucial elements in the partition mechanism.

The three critical participants of the mechanisms considered here are: (i) slow diffusion media such as a membrane surface or nucleoids (the ‘matrix’), (ii) an ATPase whose binding to the matrix is controlled through nucleotide-induced conformational changes, and (iii) a second protein component that controls the ATPase activity. Together, with possible participation of additional players (such as *parS* for P1 partition), the system generates dynamic instability in the local concentrations of the ATPase bound to the matrix and also that of the ATPase controller. This instability drives the oscillation or movement of some sort, which could be used for a cargo-carrying function, such as plasmid DNA for segregation. When certain conditions are met, the dynamic instability may cease and is replaced by a steady-state non-equilibrium spatial distribution of the components that counteracts free diffusion.

In an earlier reaction-diffusion model proposed for the Min system by Meinhardt and deBoer (2001), a key requirement was that protein molecules disappear from the system when membrane-bound MinD dissociates from the membrane with (or without) reacting with MinE. Newly synthesized proteins would then appear evenly throughout the reaction volume. However, MinD oscillation does not require ongoing protein synthesis (Raskin and deBoer, 1999), and subsequent models suggested other events, such as nucleotide exchange, might be responsible for a time delay in reactivation of the form of MinD necessary for membrane binding (Huang *et al*., 2003; Drew *et al*., 2005). Our results provide a biochemical basis for the time-delayed matrix re-binding. When protein dissociates from the matrix via ATP hydrolysis, a significant time delay before the ATPase regains its matrix-binding activity (as reported here for P1 ParA) would mean that active protein disappears from the system as described in the Meinhardt–deBoer differential equations. As long as the delay time is sufficient to allow diffusion of the molecule throughout the cell (which takes approximately 1 s; Elowitz *et al*., 1999), active molecules would ‘re-appear’ with essentially equal probability throughout the reaction volume. We believe our results are the first experimental demonstration of the time-delayed activation of a partition ATPase family member for matrix binding.

### A model for plasmid movement on the bacterial nucleoid

We propose a variation of the reaction-diffusion mechanism, which we call the diffusion-ratchet mechanism, as a possible model for partition reactions. The slow regeneration of the DNA binding form of ParA after ATP hydrolysis produces a biochemical delay that allows the nucleoid-dissociated ParA to become evenly distributed throughout the cytosol before it regains competency to bind the nucleoid. Thus, unlike the commonly considered Turing-style reaction-diffusion models, which depends on the development of inhomogeneity of the component concentrations in the cytosol and a diffusion-based time delay for the system dynamics, our model is insensitive to minor changes of the diffusion coefficients of the components in the cytosol, and the system could operate with a spatially homogeneous concentration of the cytosolic reaction components. The system dynamics depends on the kinetic properties of the biochemical steps with strategically positioned ATP hydrolysis-coupled pseudo-irreversible steps in combination with the mechano-dynamic properties of the nucleoid and the partition complex (see Ivanov and Mizuuchi, 2010 for further discussion on the related subjects).

**Fig. 7:**
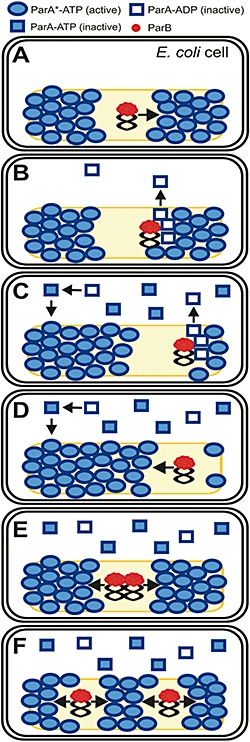
A model for diffusion-ratchet motion of a plasmid along the bacterial nucleoid.A. On the nucleoid (yellow rectangle), ParA*_2_–ATP_2_ is activated to dynamically bind DNA. ParB loads onto the plasmid (black squiggle) at *parS* forming the partition complex.B. ParB stimulates ParA ATPase activity, which clears ParA from the nucleoid in the vicinity of the partition complex. Drift of the partition complex to one direction commits its movement to continue in the same direction because of the low ParA concentration in its wake.C. ParA exchanges ADP for ATP and undergoes a slow conformational change. This time delay allows ParA to randomly diffuse and re-bind the nucleoid after ParA*_2_–ATP_2_ reforms.D. At the nucleoid pole, the partition complex changes direction. The uneven distribution of ParA on the nucleoid generates plasmid movement.E. After plasmid replication, the closely positioned partition complexes develop repulsive interactions as they both remove ParA between them.F. Multiple partition complexes develop a trend to position themselves equidistant to each other along the nucleoid. See Movie S4 for animation.

In a cartoon of a simplified diffusion-ratchet model of plasmid motion that incorporates the time-delayed ParA–DNA re-binding (Fig. 7, Movie S4), we divide ParA into essentially two states –‘active’ (DNA-binding competent) and ‘inactive’ (non-DNA binding). Most active ParA would be bound to DNA and its diffusion would be restricted. Next, we introduce the P1 plasmid partition complex to the system. As shown by *in vivo* studies (Erdmann *et al*., 1999; Rodionov and Yarmolinsky, 2004), this complex contains many ParB molecules centred around the plasmid *parS* site. Because ParB interacts directly with ParA, the partition complex would have higher affinity for the nucleoid regions with higher densities of bound ParA and a large number of ParA–ParB contacts would be made there. But as ParB associates with the nucleoid-bound ParA, ATP hydrolysis is stimulated and ParA dissociates from the nucleoid. The dissociated ‘inactive’ ParA cannot re-bind DNA immediately, diffuses throughout the cell, and thus loses the positional memory of the site of dissociation. When re-activated for DNA binding by the conformational change described in this study, binding would be random across the nucleoid. This process generates a low-density area of nucleoid-bound ParA in the vicinity of the partition complex, and this area of ParA clearance would expand with local Brownian dynamics of the partition complex and the nucleoid. Now, the partition complex is located in a higher relative potential energy area with reduced ParA contacts and would therefore drift away to establish new ParA contacts at a neighbouring area where the nucleoid-bound ParA concentration is higher. This starts the movement of the partition complex along the nucleoid. The initial drift towards one stochastically chosen direction enforces the continued movement towards the same direction, as a result of the lower concentration of the nucleoid-bound ParA in its wake. Considering the model in 3D, upon reaching a nucleoid end, the complex would be forced to pivot back or wrap around the nucleoid and move towards the other end. These ParA–ParB interactions would explain the oscillatory patterns observed with a number of partition systems (Marston and Errington, 1999; Quisel *et al*., 1999; Ebersbach and Gerdes, 2004; Fogel and Waldor, 2006; Hatano *et al*., 2007; Pratto *et al*., 2008). A recent study did not observe oscillations of P1 ParA–GFP *in vivo* (Sengupta *et al*., 2009). However, the ability to detect a dynamic pattern for the bulk ParA population by cell biology techniques would depend on the relative concentrations of cytosolic and nucleoid-bound protein, the relative amount of active partition versus repressor forms of ParA, as well as the size of the ParA-depleted regions within the spatiotemporal resolution of the microscope experiments. The details of our model could easily be modified to explain *in vivo* behaviour other than full end-to-end oscillation.

### The nucleoid matrix

In our model, the nucleoid serves as a matrix upon which the plasmids, essentially as cargo, are moved by the actions of ParA and ParB. Recent real-time fluorescence microscopy has shown that ParA–GFP of pB171 plasmid can dynamically associate with the nucleoid as helical clouds, which are chased by foci containing ParB in complex with pB171 plasmid (Ringgaard *et al*., 2009). Since several Type I partition ATPases appear as bundled filaments when viewed by electron microscopy, these subcellular helical structures have been proposed to represent laterally associated protofilaments of ParA. The resulting model is one where retracting ParA filaments move plasmids by way of a pulling mechanism. Our model could explain the observed *in vivo* pB171 system dynamics without requiring processive depolymerization of a filament to act as a contractile mechanical device. We believe that association of ParAs on the nucleoid matrix could explain the helical patterns observed by *in vivo* fluorescence microscopy, particularly since the nucleoid itself has been recently reported to be helical in shape (Berlatzky *et al*., 2008). Our model does not preclude a role for polymerization of ParA in the presence of DNA, which we think is likely akin to the polymerization of Soj that has been observed to occur on DNA (Leonard *et al*., 2005), or MinD polymerization on its matrix, the membrane (Suefuji *et al*., 2002). For bacterial ParAs such as Soj, their dynamic activity on the bacterial nucleoid could aid in organization of the origin region of the chromosome (where most *parS* sites are clustered).

Previous studies found that both P1 and F plasmid DNA could be recovered (measured by Southern hybridization) in anucleate cells of *E. coli mukB* mutants (Ezaki *et al*., 1991; Funnell and Gagnier, 1995). We think this is not inconsistent with our model because *mukB* mutants have decondensed chromosomes that can be guillotined upon cell division, so it is possible that the plasmids partitioned with fragmented chromosomes in some cells. Alternatively plasmids may segregate into anucleate cells by random diffusion, especially if the efficacy of ParA dynamics is altered by the perturbed chromosome condensation and structure in *mukB* cells.

The question of what generates bidirectional plasmid segregation is still unknown, and requires a better understanding of the interaction between ParA and the partition complex. Here, our plasmid motion model could also provide a part of the mechanism involved in replicated plasmid copy separation. When the plasmid replicates and an additional complex is formed, each partition complex would generate a low-density ParA distribution region around itself. When the two partition complexes are close together, the attraction of each plasmid copy towards regions of the nucleoid with higher densities of ParA would lead them away from the low-ParA-density area between them. Thus there would be a ‘repulsive force’ that develops and pushes the plasmid copies to separate. Multiple partition complexes would develop a trend to position themselves equidistant to each other along the nucleoid (or multiple nucleoids). This could be considered as a form of ‘interference’ phenomenon, and explains why plasmids position themselves approximately equidistantly over bacterial nucleoids (Erdmann *et al*., 1999; Ho *et al*., 2002; Ebersbach and Gerdes, 2004). Our model is consistent with recent *in vivo* microscopy that shows P1 plasmids replicating and segregating at almost any position over the nucleoid region of the cell (Sengupta *et al*., 2009). Sister plasmids were shown to move rapidly apart following separation and frequently passed close to, and sometimes ‘bumped’ into other P1 plasmids in the cell. On average, the net result was even plasmid distribution along the long cell axis.

While the above consideration provides the foundation of our model, a detailed understanding of the partition complex assembled on *parS* and its interaction dynamics with nucleoid-bound ParA is needed to mathematically predict the exact system behaviour. For example, the exact timescale of ParA–ParB interaction before ParA–DNA dissociation, and other details would have a large impact on the system dynamics. We currently do not have the necessary detailed information to formulate a realistic quantitative model, similar to the current state of our understanding of the MinD/E system as discussed recently (Ivanov and Mizuuchi, 2010). We also believe that ParB plays a more intimate role in controlling ParA conformational changes and in return, the behaviour of ParB is likely to be influenced by ParA. Preliminary evidence supports that ParB not only accelerates ParA ATP hydrolysis and conformational change back to the non-DNA binding state, but it also helps in the conformational change towards the DNA binding state. Thus, a better understanding of ParA–ParB interaction dynamics is needed to further advance our understanding of the P1 partition system mechanism.

## Experimental procedures

### Proteins

Wild-type and mutant ParAs were purified by two ion-exchange chromatography steps as previously described; this purification removes bound nucleotides (Davey and Funnell, 1994; Fung *et al*., 2001). ParA–GFP was purified similarly, with modifications described in the experimental procedures in *Supporting information*.

### Reaction buffers

Buffer A: 100 mM NaCl, 10 mM MgCl_2_, 50 mM Tris-HCl pH 7.5. Buffer B: 100 mM NaCl, 5 mM MgCl_2_, 50 mM Tris-HCl pH 7.5.

### TIRF microscopy

The fluorescence microscopy was carried out essentially as described (Greene and Mizuuchi, 2002) with modifications as described in the experimental procedures in *Supporting information*. The reaction was in buffer B supplemented with 0.1 mg ml^−1^α-casein and the nucleotide cofactor as specified for each experiment.

### ATPase assays

[γ-^32^P]-ATP was purified from contaminating ^32^P_i_ prior to use with a 1 ml gel filtration (P-2 fine resin, Bio-Rad) column. Reactions were assembled manually at 23°C and at intervals, 30 µl of samples were quenched by 30 µl of a 1% SDS, 20 mM EDTA solution, and analysed by thin-layer chromatography as previously described (Fung *et al*., 2001).

### Tryptophan fluorescence assays

Steady-state measurements were performed at 23°C with the excitation at 295 ± 1.25 nm, and the emission was monitored at 320 ± 1.25 nm. Reaction components were manually mixed in buffer A 15 min prior to the measurements. Stopped-flow measurements used an AppliedPhotophysics SX20 System at 23°C in buffer B. The excitation was 287 nm ± 0.6 nm through an FF01-280/20 filter (Semrock). The emission filter was FF01-300/LP (Semrock). For all ParA stopped-flow experiments, stock ParA solution was diluted to the experimental concentration (2× reaction concentration) in buffer B 50 min prior to the first mixing experiment. All results were averages of at least four experiments.

### Light scattering experiments

SEC/MALS experiments were carried out using Wyatt Optilab-rEX/DAWN-HELEOS connected to an Agilent Technologies 1200 Series HPLC with TOSO TSK G3000PW_XL_ column (7.8 mm × 30 cm). Thirty microlitres of 30 µM ParA was pre-incubated in buffer B supplemented with 0.1 mM EDTA and 1 mM DTT, with or without 0.5 mM nucleotide for 20 min at room temperature before injection onto the column equilibrated with the same buffer and separated with the flow rate of 0.5 ml min^−1^. Data were collected and analysed by Astra V software (Wyatt) to obtain the molar mass and hydrodynamic radius based on the Zimm method and an exponential fit with single uniform species assumption. The refractive index increment (dn/dc) was set to 0.185 cm^3^ g^−1^.

### DNA binding assay

Fluorescence quenching by unmodified ParA binding to Alexa-Fluor-514-labelled DNA containing the dye at every 11 bp was measured in stopped-flow experiments in buffer B as described in the experimental procedures in *Supporting information*.
