# Supplementary material for: ATP control of dynamic P1 ParA–DNA interactions: a key role for the nucleoid in plasmid partition
Source: Mol Microbiol. 2010 Aug 18;78(1):78–91. doi: 10.1111/j.1365-2958.2010.07314.x (PMC2950902; doi:10.1111/j.1365-2958.2010.07314.x)
Supplement: Supplementary file 5 [file mmi0078-0078-SD5.pdf]

SUPPORTING INFORMATION

Movie S1 - ParA binding to surface-tethered λ DNA after preincubation at a low ParA concentration.

Movie S2 - ParA binding to surface-tethered λ DNA after preincubation at a high ParA concentration.

Movie S3 - Association-dissociation cycle of ParA to surface-tethered λ DNA.

Movie S4 - Animated model for the P1 plasmid partition mechanism.

Fig. S1 - ParA-GFP is functional *in vivo* and *in vitro*.

Fig. S2 - MANT-nucleotide binding and dissociation kinetics.

Fig. S3 - ParA and DNA concentration effects on the kinetics of ParA's conformational change

Fig. S4 - Is the ATP-induced ParA conformational change reversible?

Fig. S5 - Dynamic light scattering of ParA with and without nucleotide.

Fig. S6. Double-stranded DNA is the preferred substrate for $ParA_2$*:ATP_2.

Supplementary Experimental Procedures

Supplementary References

Movie S1. ParA binding to surface-tethered λ DNA after preincubation at a low ParA concentration. ParA was preincubated at 25 nM in the presence of 1 mM ATP for 20 min in Buffer B. The mixture was then infused into flow cell at a flow rate of 20 μL/min. The video rate is 25 times faster than the real time. The area displayed is 80 μm x 80 μm and buffer flow is from top to bottom.

Movie S2. ParA binding to surface-tethered λ DNA after preincubation at a high ParA concentration. ParA was preincubated at 10 μM in Buffer B with 1 mM ATP for 15 min, and then diluted to 25 nM ParA (with buffer B and ATP) 5 min before infusion to the flow cell (flow rate of 20 μL/min). The video rate and display area are as described in Movie S1.

Movie S3. Association-dissociation cycle of ParA to surface-tethered λ DNA. ParA was preincubated at 10 μM in Buffer B containing 1 mM ATP for 15 min, and then diluted to 50 nM ParA (by buffer B with ATP) 5 min before infusion to the flow cell. A two-inlet laminar boundary steering flow cell was used. From one inlet, buffer B containing ParA and 1 mM ATP was infused and from the other inlet, buffer B was infused. At first, flow rate of buffer B containing ParA was set at 20 μL/min and that of Buffer B was 1 μL/min, resulting in ParA association to surface-tethered λ DNA in the field of view at the central region of the flow channel. After ParA association to the DNA, flow rates of the two inlets were switched to dissociate ParA from the DNA. Flow rates from the two inlets were switched periodically to observe the association-dissociation cycle of ParA to the surface-tethered λ DNA. Video rate and display area are as described in Movie S1.

Movie S4. Animated model for the P1 plasmid partition mechanism. See Discussion for details.

Fig. S1. ParA-GFP is functional *in vivo* and *in vitro*. (A) To assess the biological function of ParA-GFP *in vivo*, stability of a *parA*$^-$ miniP1 plasmid pBEF240 (*parB*$^+$, *parS*$^+$) was assayed with wild-type ParA (from pEF4) or ParA-GFP (from pNE34) expressed in *trans* near physiological levels. Plasmid stability was measured as the percent of *E. coli* cells that retain the plasmid after overnight growth (without selection) as described previously (Fung *et al.*, 2001). ParA-GFP was as effective as wild-type ParA in complementing the *parA* mutation of the miniP1 plasmid.

Plasmid Providing ParA (*parA* allele)	miniP1 plasmid (pBEF240) retention after overnight growth (%)
pBR322 (none)	30 ± 6
pEF4 (*parA*)	98 ± 2
pNE34 (*parA-gfp*)	96 ± 3

(B) ParA ATPase activity, which is stimulated by ParB in vitro, was measured to determine whether ParA-GFP retained wild-type function following purification. The rate of ATP hydrolysis by ParA-GFP was comparable to that of wild-type ParA. In the presence of ParB, wild-type ParA ATPase activity was stimulated ~5-fold, and ParA-GFP ATPase activity ~3-fold. Therefore, the biochemical properties of wild-type ParA are retained in ParA-GFP, albeit at slightly reduced levels.

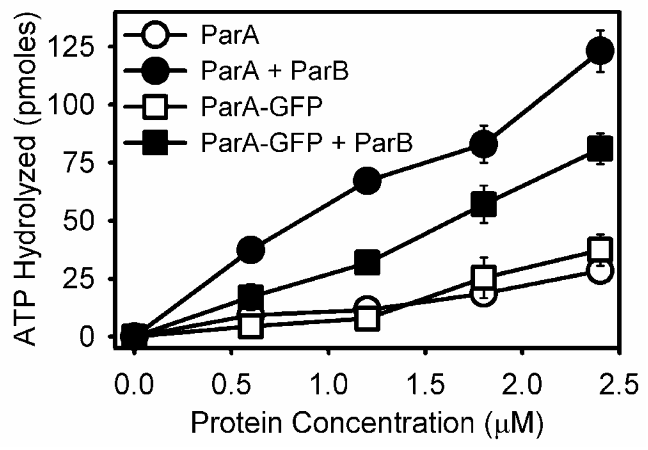

Fig. S2. MANT-nucleotide binding and dissociation kinetics. (A) ParA-MANT-ATP binding kinetics. ParA and MANT-ATP (5 μM and 10 μM respectively) were mixed rapidly in Buffer B and the change in fluorescence of MANT was monitored by stopped-flow (see Supplementary Experimental Procedures). MANT-ATP showed somewhat complex ParA association kinetics and took up to 20 seconds or longer to reach apparent steady state. The data did not fit well to a simple hyperbolic curve, in part possibly reflecting the presence of the nucleotide-free dimer population, which might display different nucleotide binding kinetics compared to the monomers. (B) ParA-MANT-ATP dissociation kinetics. MANT-ATP (10 μM) was preincubated with ParA (5 μM) for the period indicated in the figure and the dissociation kinetics were measured after rapid mixing with 1 mM ATP. The dissociation kinetics were complex, in part possibly reflecting the hydrolysis reaction; the kinetics were multiphasic and differences were observed, which depended on the time of preincubation with MANT-ATP prior to competitor addition. Longer preincubation did not cause further noticeable change in the observed kinetics (data not shown). Dissociation was complete within a few minutes, and we failed to observe the slow accumulation of a stably-bound state of ParA with MANT-ATP. We found that MANT-ATP, like other ATP-analogs, did not support ParA-DNA binding (data not shown), therefore MANT-ATP binding kinetics were not analyzed further. (C) Binding of MANT-ADP to ParA. ParA (5 μM) was mixed rapidly with MANT-ADP at the indicated concentrations, and binding was followed by stopped-flow as in (A). The extent of binding was measured at steady-state (50 sec after mixing), and the observed relative fluorescence intensity change was corrected for differences in nucleotide concentration. Half-maximum binding occurred at about 30 μM MANT-ADP indicating that ParA has similar affinity to MANT-ADP as it does for ADP, which has an apparent K_d of 50 μM (Davey and Funnell, 1997). (D) Nucleotide competition effects on ParA binding to DNA. ParA (2.5 μM) was preincubated with 0.1 mM ATP and the indicated concentration

of ADP or MANT-ADP for 3 min. The DNA-binding form of ParA generated during this period was measured by rapid mixing with Alexa-labeled DNA and following the fluorescence quenching protocol as described in Experimental Procedures (DNA binding assay). The ATP-dependent DNA binding activity of ParA was inhibited by ADP and MANT-ADP to similar extents. (E) Binding kinetics of MANT-ADP to ParA. Binding was initiated and measured as in (A), except with MANT-ADP. Like MANT-ATP, the kinetics of MANT-ADP binding to ParA were also not simple, but did clearly indicate that nucleotide binding is a slow process that takes 20 seconds or longer to reach apparent steady state. The slow nucleotide binding by ParA suggested the involvement of a protein conformational change in the binding process, such as dimerization. ParA exists in monomer-dimer equilibrium both in the presence and absence of ADP (see below), and this in part could have caused the complex kinetic behavior. (F) ParA-MANT-ADP dissociation kinetics. MANT-ADP (10 μM) was preincubated with ParA (5 μM) and the dissociation kinetics were measured after rapid mixing with 1 mM ADP. The dissociation kinetics were also complex and multiphasic with the main fraction dissociating within 20 seconds.

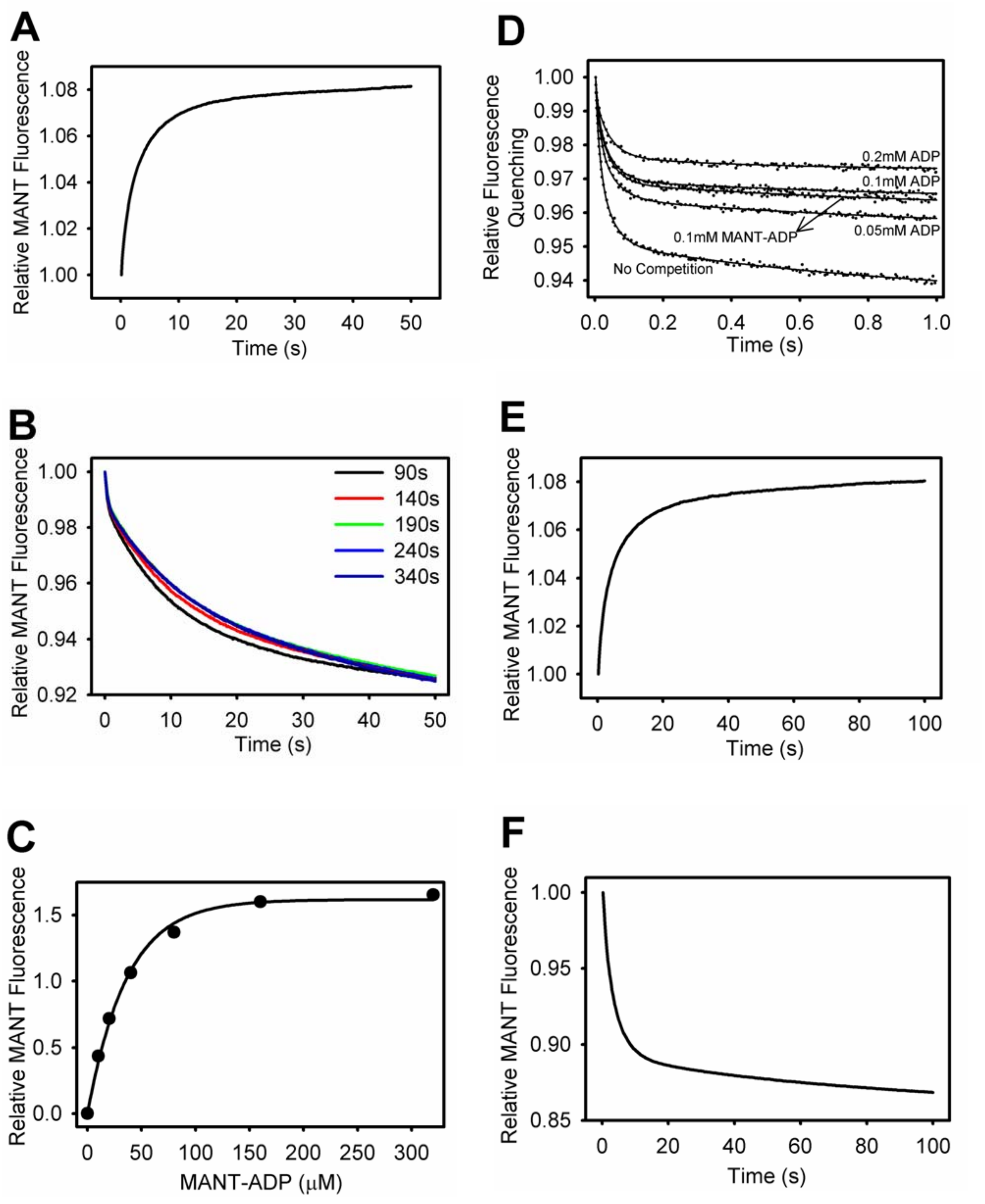
A
Relative MANT Fluorescence
Time (s)
B
Relative MANT Fluorescence
90s
140s
190s
240s
340s
Time (s)
C
Relative MANT Fluorescence
MANT-ADP (μM)
D
Relative Fluorescence Quenching
0.2mM ADP
0.1mM ADP
0.05mM ADP
0.1mM MANT-ADP
No Competition
Time (s)
E
Relative MANT Fluorescence
Time (s)
F
Relative MANT Fluorescence
Time (s)

Fig. S3. ParA and DNA concentration effects on the kinetics of ParA's conformational change as measured by tryptophan fluorescence. (A) Early phase of ParA tryptophan fluorescence change induced by ADP or ATP. The first few data points in Fig. 4D suggested an earlier event that occurs prior to ParA dimerization. Closer examination showed that there was a fluorescence decrease of ~1% in the first 0.5 sec after addition of either ADP, or to a lesser extent, ATP. Unlike the fluorescence increase, the initial fluorescence decrease was not affected by ParA concentration. We interpret this fluorescence decrease to represent the initial weak nucleotide binding of one nucleotide to ParA, which likely is not saturated at the nucleotide concentrations used in the experiments. Tryptophan fluorescence was measured as described in Experimental Procedures. (B) The kinetics of ParA's conformational change is dependent on DNA concentration. Measurements were performed in a Photon Technology International (PTI) system maintained at 23°C. The excitation wavelength was set at 295 ± 1.3 nm, and the emission was monitored at a wavelength of 320 ± 1.2 nm. In buffer A, 5 μM ParA was pre-incubated with sonicated salmon-sperm DNA at the concentrations indicated. The samples were then mixed with 1 mM ATP, and changes in tryptophan fluorescence were monitored over time. All results are an average of at least 3 independent experiments. Without DNA, ATP induced a slow change in the tryptophan fluorescence of ParA. In the presence of DNA, the fluorescence change was accelerated, and the rate of tryptophan fluorescence change was dependent on DNA concentration. With 1 mg/ml DNA, ParA reached apparent steady state in less than one minute; a rate ~ 10-fold faster than in the absence of DNA.

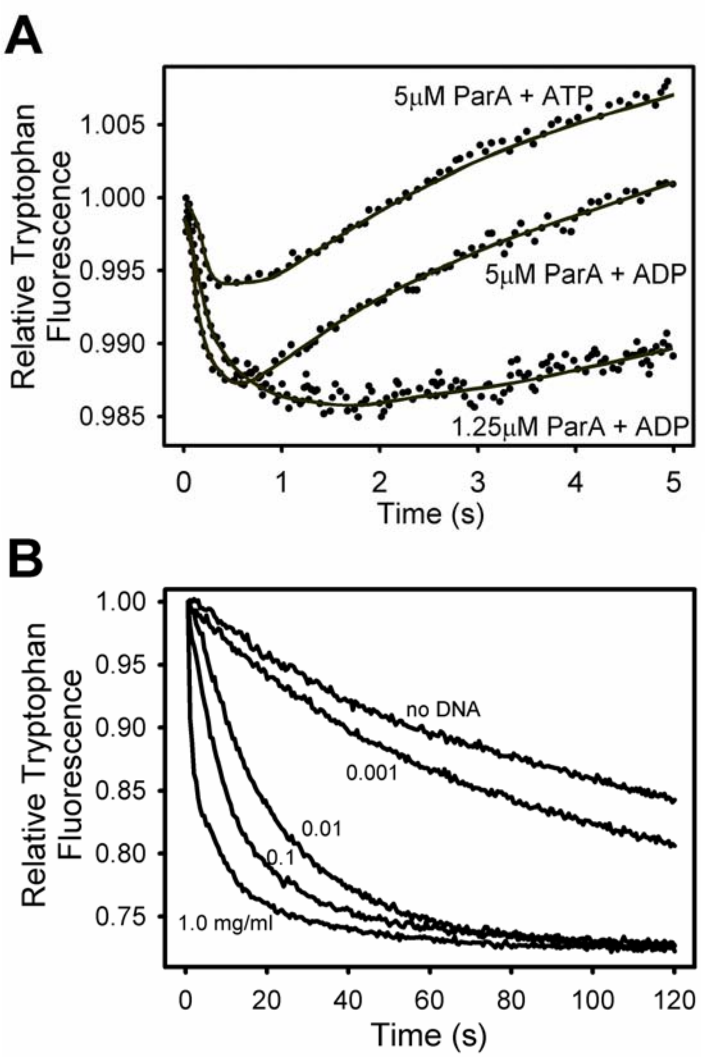
A
5μM ParA + ATP
5μM ParA + ADP
1.25μM ParA + ADP
Relative Tryptophan Fluorescence
1.005
1.000
0.995
0.990
0.985
0
1
2
3
4
5
Time (s)
B
no DNA
0.001
0.01
0.1
1.0 mg/ml
Relative Tryptophan Fluorescence
1.00
0.95
0.90
0.85
0.80
0.75
0
20
40
60
80
100
120
Time (s)

Fig. S4. Is the ATP-induced ParA conformational change reversible? (A) The ATP-induced ParA conformation change is slowly reversed by ADP. We tested whether the ATP-induced conformational change is a reversible reaction. ParA (10 μM) in buffer B underwent an approximate 29% decrease in fluorescence with 50 μM ATP (▲). When 2 mM ADP competitor was then added (arrow), ParA tryptophan fluorescence slowly increased back to the expected level of fluorescence for ParA-ADP. In the presence of 0.1 mg/ml sonicated DNA (○), the rate of conversion to ParA*-ATP increases, but there is no significant effect on the rate of reversal. The results show that ParA conformation reversal occurs at a similar rate to that of nucleotide exchange of stably bound ParA-ATP (compare with Fig. 3B). (B) Dilution does not induce ParA conformation re-equilibration. We tested whether the concentration dependence of the tryptophan fluorescence change in ParA (Fig. 4E and F) could be explained by a reversible change in ParA oligomeric state. In this case, a two-fold dilution is expected to cause a fluorescence increase of ~ 3% in 500 sec in the absence of DNA, and a 4% increase in 100 sec in the presence of DNA. These are expected fluorescence differences due to differences in the extent of conformational change at 5 μM and 2.5 μM ParA. ParA (5 μM) was preincubated in Buffer B with 0.5 mM ATP in the presence or absence of 100 μg/ml DNA for 20 min, rapidly diluted 2-fold in the same buffer (with ATP, with or without DNA), and the tryptophan fluorescence change was monitored by stopped-flow. Both in the presence and absence of DNA, dilution of ParA resulted in no conformation reversal. The slow fluorescence decrease observed mostly reflects photobleaching.

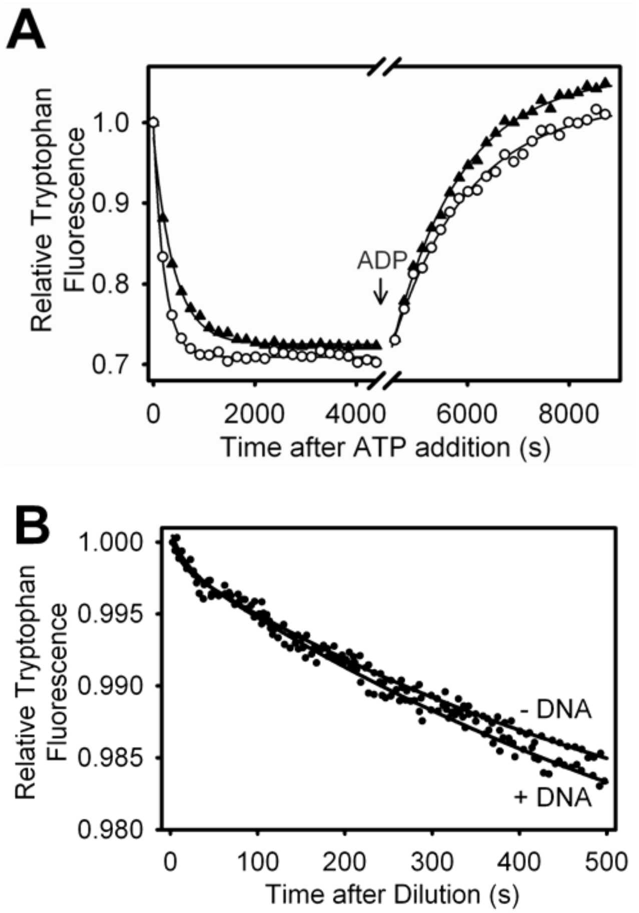
A
Relative Tryptophan Fluorescence
1.0
0.9
0.8
0.7
ADP
0
2000
4000
6000
8000
Time after ATP addition (s)
B
Relative Tryptophan Fluorescence
1.000
0.995
0.990
0.985
0.980
- DNA
+ DNA
0
100
200
300
400
500
Time after Dilution (s)

Fig. S5. Dynamic light scattering of ParA with and without nucleotide. Dynamic light scattering was measured as described in Supplementary Experimental Procedures. (A) Without nucleotide, data were well modeled in terms of a single non-interacting species having a hydrodynamic radius r_h of 3.3 nm, consistent with the presence of ParA monomers and dimers. (B) Similar observations were made in the presence of 1 mM ADP with a hydrodynamic radius r_h of 3.5 nm, again consistent with a mixture of ParA monomer and dimers. (C) In the presence of added ATP, the autocorrelation function showed the presence of very small amounts of larger aggregates. Analysis in terms of two non-interacting species returned an r_h of 3.1 nm, whereas a single solute analysis returned a value of 3.9 nm (shown). Together, these data show that in the presence of ATP the major ParA species are presumably monomers and dimers.

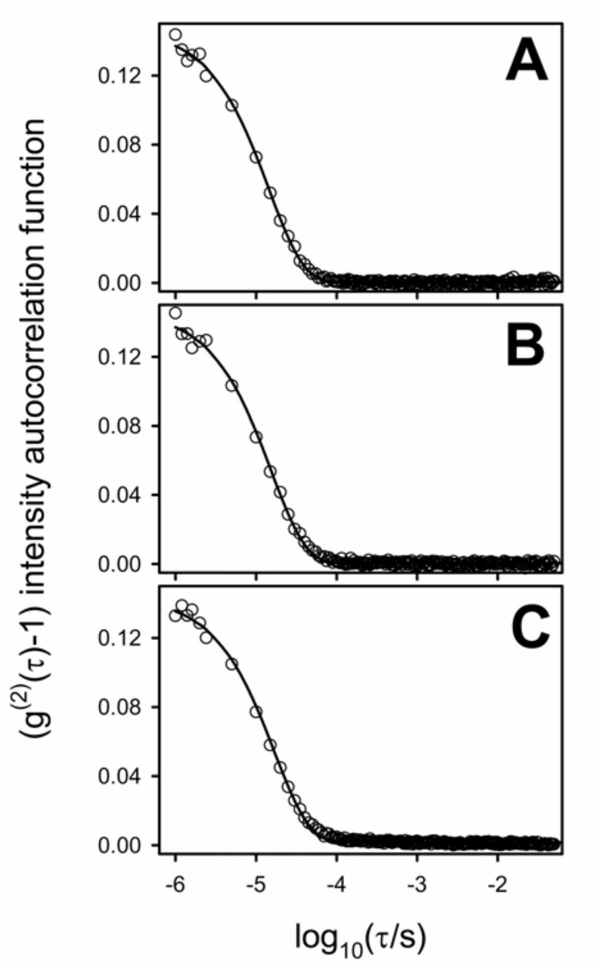

Fig. S6. Double-stranded DNA is the preferred substrate for ParA$_2$*:ATP$_2$. We propose that ParA's binding to non-specific double-stranded DNA (dsDNA) *in vitro* reflects its nucleoid binding activity *in vivo*. This predicts that RNA and single-stranded DNA (ssDNA) should be poor substrates for ParA$_2$*:ATP$_2$. We measured the ability of dsDNA, ssDNA or RNA to compete with fluorescently labeled dsDNA for ParA binding. ParA (2.5 μM) was preincubated with 1 mM ATP for 15 min and rapidly mixed with an equal volume of 25 μg/ml fluorescent dsDNA with or without 100 μg/ml competitor (sonicated salmon sperm dsDNA, ϕX174 ssDNA, or RNA purified from *E. coli*), and DNA binding was measured by fluorescence quenching (see Supplementary Experimental Procedures). Unlabelled dsDNA was the only strong competitor for ParA binding to the labeled dsDNA substrate. ssDNA showed slight competition, which could be attributed to the secondary structure of the ssDNA substrate. RNA showed no competition.

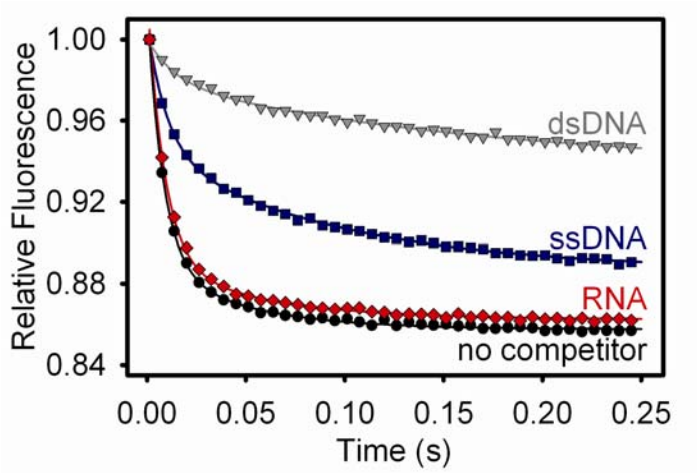

Supplementary Experimental Procedures

***Escherichia coli* strains, media and plasmids.** Plasmids were maintained in the *E.coli* K12 strain DH5 [F^- *endA1 hsdR17* (rk^-mk^+) *supE44 thi-1 recA1 gyrA96 relA1*]. All bacterial cells were grown in LB medium or on LB plates. Plasmid pEF4 contains the *parA* gene under control of a modified β-lactamase promoter (*blaP*$_1$; Fung *et al.*, 2001). For *parA-gfp*, the *gfp(mut3.1)* gene was inserted 3' of the *parA* gene of pEF4, and included the linker sequence 5'AAAGAGGAGAAATTAAGC (removing the C-terminal Asn of ParA and inserting the 6 residue linker KEEKLS between ParA and GFP of the protein), creating pNE34. The plasmid used for ParA-GFP protein production (pAV12) contains the *parA-gfp* gene under the control of a bacteriophage T7 promoter in the vector pET15b (Novagen). The miniP1 plasmid used for stability analyses, pBEF240, contains a 4 bp insertion (at the *Xho*I site) in the *parA* gene of pLG49 (Funnell and Gagnier, 1995).

ParA-GFP purification. ParA-GFP was purified essentially as described for wild-type ParA with modifications: Protein expression was induced by IPTG at 20°C for 6-8 hours. Following the S-sepharose chromatography step, a 1ml HiTrap Q HP (GE Healthcare) column was equilibrated with Q buffer containing 140 mM KCl, and bound protein was eluted with a linear 140 mM to 1 M KCl gradient.

Total Internal Reflection Fluorescence Microscopy. The TIRF microscope was built around a Nikon TE 2000 E Eclipse frame (Nikon). A 488-nm diode-pumped, solid-state laser (Coherent) was focused through a fused silica prism onto a flow cell containing the immobilized DNA. Fluorescent images were formed through an objective lens (100x Plan Apo VC, NA 1.4, Nikon) and a tube lens and captured by a CCD (Cascade II 512, Roper Scientific) through notch filters (NF-01-488/532/635-

25x5.0, Semrock) using Metamorph 6 imaging software (Molecular Devices). The flow cells were assembled as described previously (Tan *et al*, . 2007). Before use, the flow cells were first incubated for 30 min at room temperature with Buffer TN100 (10 mM Tris-HCl pH 8.0, 100 mM NaCl) containing 1 mg/mL Liposome (SUV was prepared by sonication of multilamellar vesicles prepared with DOPC mixed with 1% biotin-PE; Avanti Polar Lipids), then rinsed with Buffer TN100, followed by incubation for 30 min at room temperature with Buffer TN100 containing 1 mg/ml Neutravidin (Pierce). The flow cells were then rinsed with Buffer B containing 2 mg/ml α-casein to ensure blocking of the exposed glass/fused silica surfaces. The biotinylated λ DNA was infused into the flow cells using a syringe pump at 50 μL/min for 10 min and incubated for an additional 10 min to immobilize the DNA. Buffer B supplemented with 0.1 mg/ml α-casein was pumped into the flow cell at 50 μL/min for 10 min to wash out unimmobilized DNA.

MANT-nucleotide binding and dissociation assays. Kinetic measurements with MANT (*N*-methylanthraniloyl)-nucleotides (Molecular Probes) were performed at 23°C using a AppliedPhotophysics SX20 System. The excitation monochrometer was set to 356 nm ± 1.2 nm, and an FF01-355/40 filter (Semrock) was inserted to the light path to reduce the background. The emission filter on the PMT (Hamamatsu R6095) was FF01-BLP-405R (Semrock). The stock ParA solution was diluted to the experimental concentration (2x reaction concentration) in buffer B 50 min prior to the first mixing experiment. All results are average of at least four experiments. Values are reported as relative fluorescence increase or decrease.

Dynamic light scattering. The translational diffusion coefficient D was measured from autocorrelation analysis of the quasielastically scattered light. The λ = 514.5 nm emission of an argon

ion laser (Lexel, Model 95) was used in the TEM_{00} mode with an output power adjusted to 180 mW. Data were collected at 90° on a Brookhaven Instruments BI-200SM goniometer with the photomultiplier tube aperture set to 400 μm. Autocorrelation functions were collected at 21.0°C with a Brookhaven Instruments BI-9000 AT autocorrelator and sampling times τ of 0.5 μs to 50 ms.

ParA samples were further purified by gel filtration through a Superdex 200 column and prepared in buffer B. 180 μL of ParA (10 μM) was transferred to a cuvette and autocorrelation functions were accumulated for 5 to 10 minutes. ADP or ATP was added to a final concentration of 1 mM, as indicated at least 10 min prior to the data collection. In all cases samples were spun at 10,000 rpm at 20ºC for 5 minutes prior to data collection in order to remove dust and other large particulate matter. Autocorrelation functions were imported into SEDFIT 11.8 (Schuck, 2009). The program was downloaded from http://www.analyticalultracentrifugation.com/sedfit.htm and analyzed in terms of a single diffusing species. Buffer viscosities were obtained based on the composition using SEDNTERP (Cole *et al*., 2008). The program was downloaded from http://www.jphilo.mailway.com/download.htm.

DNA binding assay. Oligos used for DNA binding assay were Y014, 5'-T*GA GTG TGT AAA TTT TAA TTT A and Y015, 5'-T*TA CAC ACT CAT AAA TTA AAA T. Both were phosphorylated at the 5' ends and "T*" at the 5' ends stands for amino-modified thymine. Amino-modified oligos were fluorescence labeled using Alexa-514 carboxylic acid, succinimidyl ester (Invitrogen) and purified according to the protocol of the manufacturer. Two 22 nt oligos, complementary to each other by a 11 nt stagger, were mixed in a buffer (50 mM Tris-HCl pH 7.5, 10 mM MgCl2, 1 mM ATP and 10 mM DTT), annealed overnight and ligated to generate poly-dispersed length fragments of a few kbp with sequence repeats containing a dye at every 11 bp. The reaction mixture was incubated at 65 ºC for 20

min to inactivate ligase, and the buffer was changed to TE using a spin column. All kinetic measurements were carried out using a stopped-flow spectrafluorometer (Model SX20; Applied Photophysics). The emission filter used was Semrock FF01-542/41-25. Excitation was at 495 nm. The stock ParA solution was diluted to the experimental concentration (2x reaction concentration) in buffer B 50 min prior to the first mixing experiment. All results were averages of at least four experiments.

Supplementary References

Cole, J. L., Lary, J. W., Moody, T, P., and Laue, T. M. (2008). Analytical ultracentrifugation: sedimentation velocity and sedimentation equilibrium. *Methods Cell Biol* 84: 143-79.

Schuck, P. (2000) Size-distribution analysis of macromolecules by sedimentation velocity ultracentrifugation and Lamm equation modeling. *Biophysical J* **78**: 1606 - 1619.

Tan X., Mizuuchi M., and Mizuuchi K. (2007) DNA transposition target immunity: Determinants of the MuB distribution patterns on DNA. *Proc Natl Acad Sci USA* **104**: 13925-13929.
